# Roth Spots As the Initial Manifestation of Accelerated-Phase Chronic Myeloid Leukemia

**DOI:** 10.7759/cureus.107301

**Published:** 2026-04-18

**Authors:** Meryem Benchekroun, Salma Hamidi, Bouchra Fakhir, Saad Benchekroun Belabbas, Lalla ouafa Cherkaoui

**Affiliations:** 1 Ophtalmology A, Specialty Hospital of Rabat, Mohamed V University, Rabat, MAR; 2 Medicine, University Hospital Ibn Sina, Rabat, MAR

**Keywords:** chronic myeloid leukemia (cml), fundus examination, hematologic malignancy, retinal hemorrhage, roth spots

## Abstract

Roth spots are white-centered retinal hemorrhages associated with various systemic conditions, particularly infective endocarditis and hematologic diseases. We report a case in which Roth spots were the presenting sign of accelerated-phase chronic myeloid leukemia. A 54-year-old man with no significant medical history presented to the ophthalmology emergency department with acute decreased vision in the left eye associated with fever and generalized weakness for four weeks. Fundus examination revealed multiple bilateral white-centered retinal hemorrhages consistent with Roth spots, associated with mild vitreous hemorrhage in the left eye. Laboratory investigations revealed severe anemia, marked leukocytosis, and thrombocytopenia. Peripheral blood smear and bone marrow examination confirmed the diagnosis of accelerated-phase chronic myeloid leukemia. The patient was referred to hematology and started on systemic treatment. This case highlights the importance of fundus examination in detecting systemic hematologic diseases and emphasizes that Roth spots may be the first manifestation of chronic myeloid leukemia.

## Introduction

Roth spots are retinal hemorrhages with a white or pale center, first described by Moritz Roth in 1872. They were initially considered pathognomonic of infective endocarditis but are now recognized as a non-specific sign associated with multiple systemic conditions, including leukemia, anemia, thrombocytopenia, hypertensive retinopathy, diabetic retinopathy, and connective tissue diseases [1,2].

The white center of Roth spots is believed to represent fibrin-platelet aggregates, inflammatory infiltrates, or ischemic retinal tissue resulting from retinal capillary rupture [3]. Although not specific to any disease, their presence should prompt systemic evaluation because they may be the first sign of a serious systemic disorder [4].

Chronic myeloid leukemia (CML) is a myeloproliferative neoplasm characterized by uncontrolled proliferation of myeloid cells due to the breakpoint cluster region-Abelson murine leukemia viral oncogene homolog (BCR-ABL) fusion gene [5]. Ocular manifestations of leukemia may include retinal hemorrhages, cotton wool spots, vascular occlusions, ischemic retinopathy, and vitreous hemorrhage [6]. In some cases, ocular findings may be the first manifestation of the disease [7].

We report a case in which Roth spots were the presenting sign leading to the diagnosis of accelerated-phase chronic myeloid leukemia.

## Case presentation

A 54-year-old man with no known medical history presented to the ophthalmology emergency department with acute decreased vision in his left eye. The patient first developed systemic symptoms, including fever and generalized weakness, four weeks before presentation, for which he self-medicated with paracetamol. This was followed by a progressive decrease in visual acuity in the left eye in the days preceding consultation.

**Figure 1:**
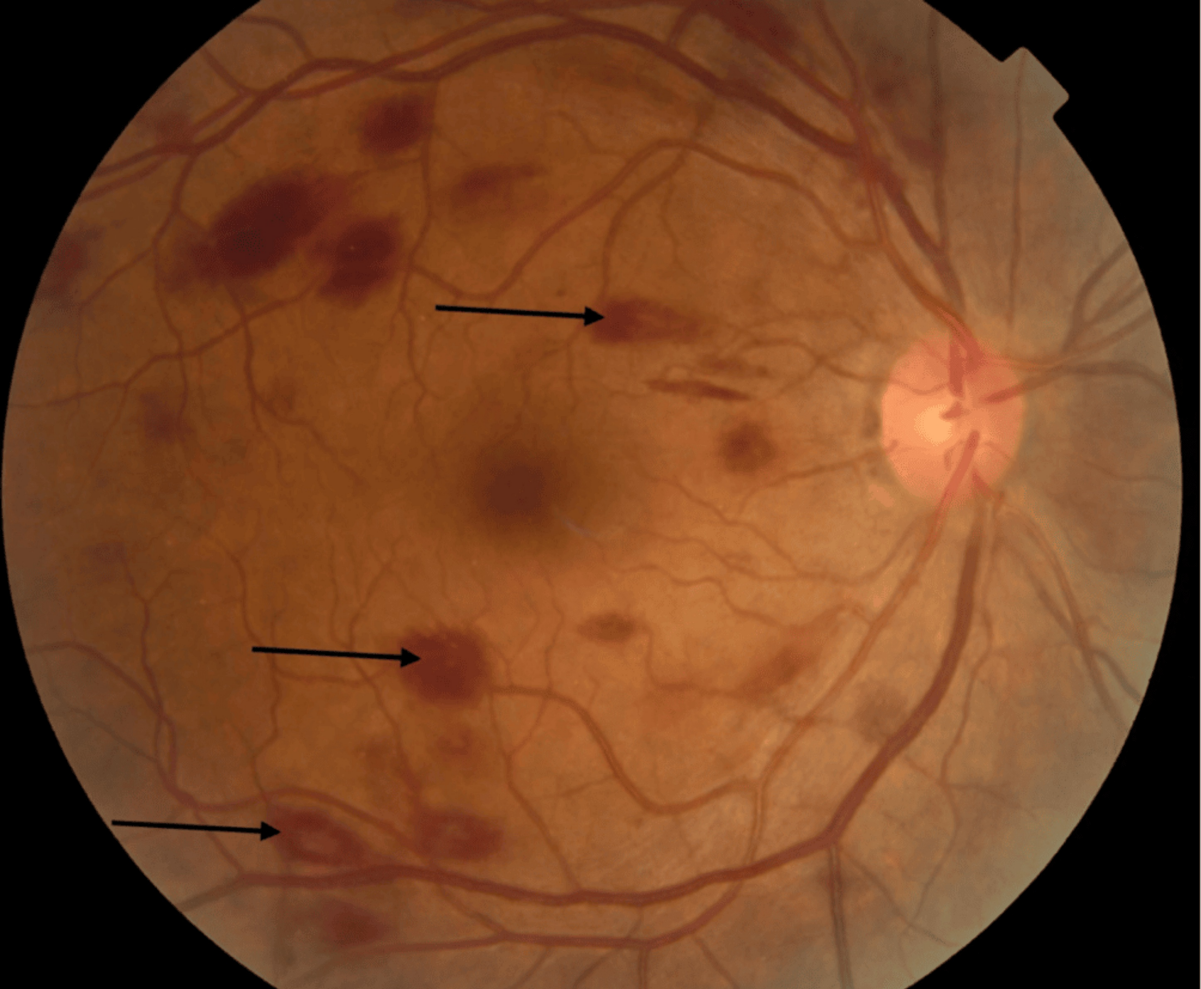
Fundus photograph of the right eye showing multiple white-centered retinal hemorrhages (Roth spots) associated with flame-shaped retinal hemorrhages. Color fundus photograph of the right eye showing multiple intraretinal hemorrhages distributed over the posterior pole and along the vascular arcades. The black arrows indicate white-centered retinal hemorrhages (Roth spots), characterized by retinal hemorrhages with pale centers corresponding to fibrin-platelet aggregates, leukemic infiltration, or ischemic debris. The presence of multiple Roth spots suggested an underlying systemic disorder, particularly a hematologic disease, which prompted further systemic investigation.

**Figure 2:**
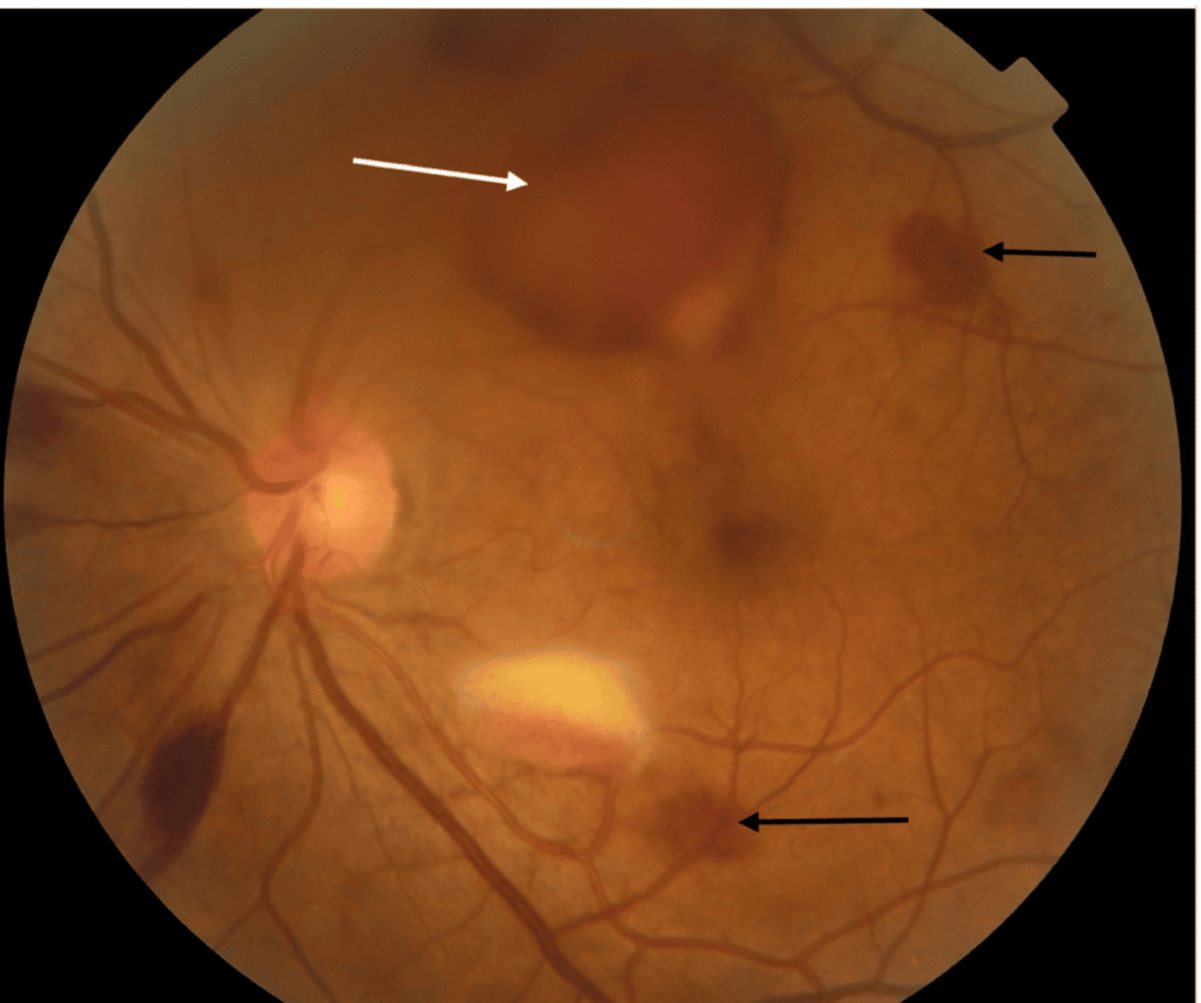
Fundus photograph of the left eye showing a large white-centered retinal hemorrhage associated with multiple intraretinal hemorrhages and mild vitreous hemorrhage. Color fundus photograph of the left eye demonstrating multiple white-centered retinal hemorrhages (Roth spots) indicated by black arrows. The white arrow indicates a mild vitreous hemorrhage partially obscuring the posterior pole, which explains the decreased visual acuity in the affected eye. These ocular findings were the initial manifestation that led to systemic evaluation and the diagnosis of accelerated phase chronic myeloid leukemia.

Best-corrected visual acuity was counting fingers at 0.5 meters in the left eye and 4/10 in the right eye. Slit-lamp examination of the anterior segment was normal in both eyes. Fundus examination revealed multiple (more than 10) bilateral white-centered flame-shaped retinal hemorrhages consistent with Roth spots, predominantly distributed over the posterior pole and along the vascular arcades, associated with mild vitreous hemorrhage in the left eye (Figures 1, 2).

The patient was referred for systemic evaluation. Hemodynamic status was stable, and transthoracic echocardiography showed no evidence of infective endocarditis.

Laboratory investigations revealed severe anemia with hemoglobin of 6.4 g/dL, leukocytosis with white blood cell count of 132,760/mm³, and thrombocytopenia with platelet count of 42,000/mm³. Peripheral blood smear showed a predominance of myeloblasts (72%). Bone marrow examination revealed 18% blasts and 40% basophils, consistent with accelerated phase chronic myeloid leukemia. The patient was referred to the hematology department, and systemic treatment was initiated. He is currently under hematologic follow-up.

## Discussion

Roth spots are white-centered retinal hemorrhages that represent a non-specific ophthalmologic finding associated with various systemic diseases. Although historically linked to infective endocarditis, they are now frequently associated with hematologic disorders, including leukemia, anemia, and thrombocytopenia [1,2].

The pathophysiology of Roth spots involves rupture of retinal capillaries followed by platelet-fibrin thrombus formation at the site of vascular injury. Histopathological studies have demonstrated that the white center corresponds to fibrin, platelet aggregates, inflammatory infiltrates, or ischemic retinal tissue [3,4].

Ocular manifestations are common in leukemia and may occur in a significant proportion of patients during the course of the disease. Retinal hemorrhages are the most frequent ocular finding and are related to anemia, thrombocytopenia, hyperviscosity, and leukemic infiltration of retinal vessels [5,6]. Other ocular manifestations include cotton wool spots, papilledema, central retinal vein occlusion, and vitreous hemorrhage [6,7].

Chronic myeloid leukemia progresses through three phases: chronic phase, accelerated phase, and blast crisis. Accelerated phase is characterized by increased blasts, basophilia, thrombocytopenia, and disease progression [8,9]. Ocular manifestations in chronic myeloid leukemia are mainly related to hematologic abnormalities and hyperviscosity syndrome, leading to retinal hemorrhages and ischemic changes [6,7].

Several case reports have described Roth spots as the initial manifestation of leukemia, emphasizing the important role of ophthalmologists in detecting systemic diseases [10,11]. Fundus examination may therefore contribute to early diagnosis and management of life-threatening hematologic malignancies [12].

In our case, Roth spots were the first clinical sign that led to the diagnosis of accelerated-phase chronic myeloid leukemia, highlighting the importance of ophthalmologic examination in systemic disease diagnosis.

## Conclusions

Roth spots are an important but non-specific ophthalmologic sign that may indicate serious systemic disease, particularly hematologic malignancies and infective endocarditis. Their presence should prompt immediate systemic evaluation, including complete blood count and infectious workup. This case illustrates that Roth spots may be the initial manifestation of accelerated-phase chronic myeloid leukemia and emphasizes the important role of ophthalmologists in the early diagnosis of systemic diseases.
